# Maternal genomic profile, gestational diabetes control, and Mediterranean diet to prevent low birth weight

**DOI:** 10.1016/j.isci.2024.111376

**Published:** 2024-11-13

**Authors:** Ana M. Ramos-Levi, Rocío Martín O'Connor, Ana Barabash, Maria Paz de Miguel, Angel Diaz-Perez, Clara Marcuello, Cristina Familiar, Inmaculada Moraga, Maria Arnoriaga-Rodriguez, Johanna Valerio, Laura del Valle, Veronica Melero, Mirella Zulueta, Leire Mendizabal, María Jose Torrejon, Miguel Angel Rubio, Pilar Matia-Martín, Alfonso Calle-Pascual

**Affiliations:** 1 Department of Endocrinology and Nutrition, Hospital Clínico Universitario San Carlos and Instituto de Investigación Sanitaria del Hospital Clínico San Carlos (IdISSC), Madrid, Spain. Calle Profesor Martin Lagos s/n, 28040 Madrid, Spain; 2 Facultad de Medicina. Medicina II Department, Universidad Complutense de Madrid, Spain. Av. Complutense, 28040 Madrid, Spain; 3 Centro de Investigación Biomédica en Red de Diabetes y Enfermedades Metabólicas Asociadas (CIBERDEM), Madrid, Spain; 4 Patia Europe, Clinical Laboratory, Paseo Mikeletegi 69, San Sebastián, Spain

**Keywords:** Clinical genetics, Female reproductive endocrinology

## Abstract

Low birth weight (LBW) is associated to poor health outcomes. Its causes include maternal lifestyle, obstetric factors, and fetal (epi)genetic abnormalities. This study aims to increase the knowledge regarding the genetic background of LBW by analyzing its association with a set of 110 maternal variants related to gestational diabetes mellitus, in the setting of a nutritional intervention with Mediterranean diet. The analysis follows a multifactorial approach, including maternal genetic information of 1,642 pregnant women, along with their anthropometric and metabolic characteristics. Binary logistic regression models provided 33 discovery variants associated with LBW that underwent a functional enrichment process to obtain a protein/gene interaction network and 126 enriched terms. Overall, our analysis proves that genetic variants form proximity clusters, grouped into subsets statistically associated with underlying biological processes or other maternal characteristics, which, on their part, allow early prevention of the eventual risk of LBW.

## Highlights

• Reducing the prevalence of LBW is a target endorsed by the World Health Assembly
• Maternal SNPs and lifestyle impact the occurrence of LBW
• Genetic variants associated with LBW form clusters related with maternal phenotypes
• LBW is prevented with an appropriate diet and modus vivendi during pregnancy

## Graphical abstract

**Figure:**
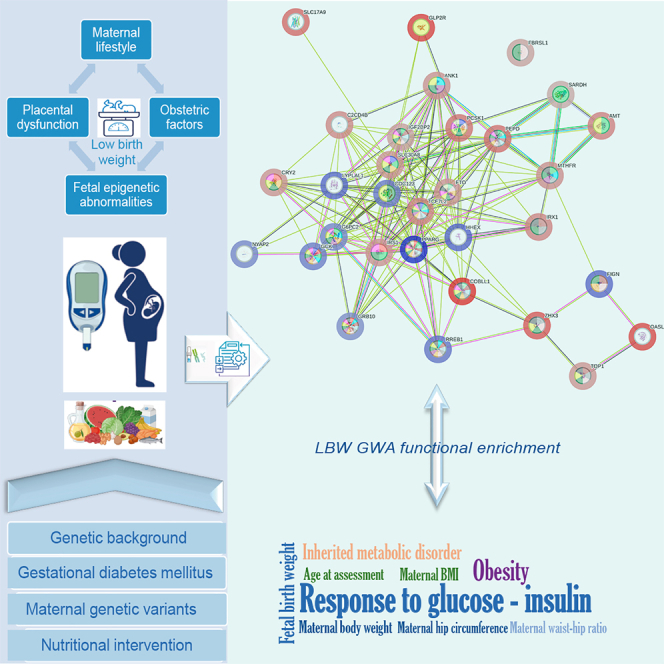

## Introduction

Birth weight of a newborn is an important biomarker used as an indicator of fetal health and nutrition. Its measurement is recommended immediately after delivery, before the neonatal early-days’ weight loss occurs. An excessively low or high birth weight is statistically associated with adverse health outcomes for the newborn. Specifically, babies born with low birth weight (LBW) have a higher risk of stunting, lower IQ, and even death during childhood, while it can cause overweight and obesity, diabetes, and heart disease during adulthood.^1^ The World Health Organization (WHO) includes LBW as a primary outcome indicator in the core set of indicators for the Global Nutrition Monitoring Framework and includes it in the WHO Global reference list of 100 core health indicators. In this regard, the WHO has established a threshold of 2500 g (5.5 lbs) to define a newborn as LBW worldwide.^1^ Statistics published by the United Nations Children’s Fund (UNICEF) and WHO, corresponding to the period 2000–2015, show that the prevalence of LBW ranges between 7.2% and 17.3% in the different United Nations regions and sub-regions, reaching 14.7% worldwide.^1^ These data are approximately maintained in the estimates that appear on the UNICEF-WHO website updated as of July 2023.^2^ Reducing the prevalence of LBW by at least 30% between 2012 and 2025 is a target endorsed by the World Health Assembly that can contribute to achieving Sustainable Development Goal 2 (Zero Hunger) by 2030.^3^

The WHO’s definition of LBW is objective and easy to calculate, although it does not consider gestational age, sex assigned at birth, or factors such as intrauterine growth retardation (IUGR), which may influence fetal growth. On another part, maternal influences on fetal growth are determined by nutrient intake, health conditions, medication, habits, and genetic factors. This means that biological and pathological conditions can interfere with growth potential and reduce size at birth.

In the clinical management of abnormal fetal growth, the expression small-for-gestational-age (SGA) is used to designate newborns with a birth weight and/or length below the normal range for gestational age. The recent recommendations of the International Consensus Guideline on Small for Gestational Age define SGA as being born with birth weight and/or length below −2 SDS (Standard Deviation Score) for gestational age according to national reference standards.^4^ The SGA definition is complex because it requires accurate knowledge of gestational age, precise anthropometric measurements at birth, and appropriate reference data for birth weight and birth length.^4^^,^^5^^,^^6^

Causes of LBW are multifactorial and include maternal lifestyle and obstetric factors, placental dysfunction, and numerous fetal (epi)genetic abnormalities. Fetal growth and weight gain are complex balanced process in which demands of the fetus and maternal placenta interact, ideally without harm to the mother’s health. One of the key regulators of fetal growth is insulin. As such, in women with diabetes, the fetus gains weight in response to insulin hypersecretion secondary to maternal hyperglycemia, and not so much due to an increase in the transfer of nutrients from the placenta *per se*.^7^ This explains why both type 2 diabetes (T2D) and gestational diabetes mellitus (GDM) have been previously associated with differences in birth weight.^8^^,^^9^^,^^10^^,^^11^^,^^12^^,^^13^ In this regard, GDM, defined as diabetes newly diagnosed in the second or third trimester of pregnancy that was not clearly overt diabetes prior to gestation,^14^^,^^15^ is a frequent gestational metabolic complication that has become a major public health issue. Its prevalence has significantly augmented in parallel with increasing rates of obesity and older age at pregnancy. GDM is associated with adverse maternal and neonatal outcomes, including birth weight deviations,^14^^,^^15^ and has a relevant genetic basis that has been revealed in various investigations.^16^^,^^17^^,^^18^^,^^19^

The association between birth weight and the genomic profile of both the mother and her offspring has been evaluated in several recent publications. These studies focus on analyzing the influence of genetic variants in birth weight, considering their capacity to predict specific adult disorders, such as cardio metabolic risk, and T2D.^20^^,^^21^^,^^22^^,^^23^ It is interesting to note that some results show that the maternal genetic profile provides relevant information, regardless of that obtained by studying the offspring’s own genetic profile.^20^^,^^23^^,^^24^ Similarly, there are interesting reports on the clinical treatment of growth retardation, studying the association of genetic variants with newborn biomarkers, such as birth weight, height and head circumference, which characterize certain monogenic disorders related to short stature.^4^^,^^25^

Beyond the clinical treatment of fetal growth restriction and birth weight, this study aims to investigate the early prevention of LBW newborns, based on their mothers’ genetic profile, in a multinational and multiethnic sample. Given that The Monarch Initiative^26^ and The Human Phenotype Ontology^27^ use the code HP:0001518 to designate the phenotypic feature that includes as synonyms the terms LBW and SGA, in this study, we will use the experimental variable directly related to the baby’s weight phenotype, since it provides adequate information for the purpose of this work.

This study forms part of a broader project initiated in 2015 in the Endocrinology and Nutrition Department of the San Carlos Clinical Hospital in Madrid (Spain), which is a public university hospital and healthcare institution that is responsible for monitoring pregnancy, childbirth, and postnatal care of women belonging to a group of nearly half a million people, whose sociodemographic characteristics may be considered as representative of the population currently living in the country. The general objective of the project is the prevention and control of GDM, with special emphasis in a context of a nutritional intervention (NI) with a Mediterranean diet (MedDiet). It has been developed in various phases, including a randomized controlled trial (RCT), registered December 4, 2013, at SRCTN84389045 (DOI 10.1186/ISRCTN84389045), and a real-world study, registered October 11th, 2016, at ISRCTN13389832 (DOI 10.1186/ISRCTN13389832), both with approval by the Clinical Trials Committee of the Hospital Clínico San Carlos (July 17, 2013, CI13/296-E and October 1st, 2016, CI16/442-E, respectively), and compliance with the Declaration of Helsinki. Consequently, we have a real data warehouse with data collected in everyday clinical practice that we call the San Carlos Cohort. Research conclusions derived from these data warehouse have been previously published in several papers.^28^^,^^29^^,^^30^

This study aims to contribute to increasing the knowledge regarding the genetic background of LBW by analyzing its association with a set of maternal genetic variants related to GDM. Our approach to the genetic factors that influence newborn LBW will consist on discovering and evaluating the eventual association between this phenotype and several maternal single nucleotide polymorphisms (SNPs), to promptly identify maternal genetic profiles that may influence the trait and, consequently, enhance preventive recommendations to guide future mothers.

As a starting point, 110 SNPs were selected for their relationship with T2D and/or GDM, according to results of large meta-analysis of genome-wide association studies (GWASs) performed in European and other populations, with the presumption that their effects can be extrapolated and generalized, and that large sample sizes allow solid estimations of the true effect.

Data regarding clinical, demographic, and anthropometric characteristics was collected from medical records and follow-up visits. Specifically, for the objectives of this study, we postulated that significance and effect size of association test between variants and LBW phenotype can be modified by the following factors, biometric, and metabolic characteristics of pregnant woman: ethnicity, present status of GDM, NI group, age, height, weight, body mass index, glucose, and insulin at the time of the oral glucose tolerance test (OGTT).

The main objectives of this work will be to analyze several association statistical hypotheses that translate the different models of genetic inheritance and include variables that are postulated as possible modifiers of the sought association.

## Results

### Patient characteristics and LBW

**Figure 1:**
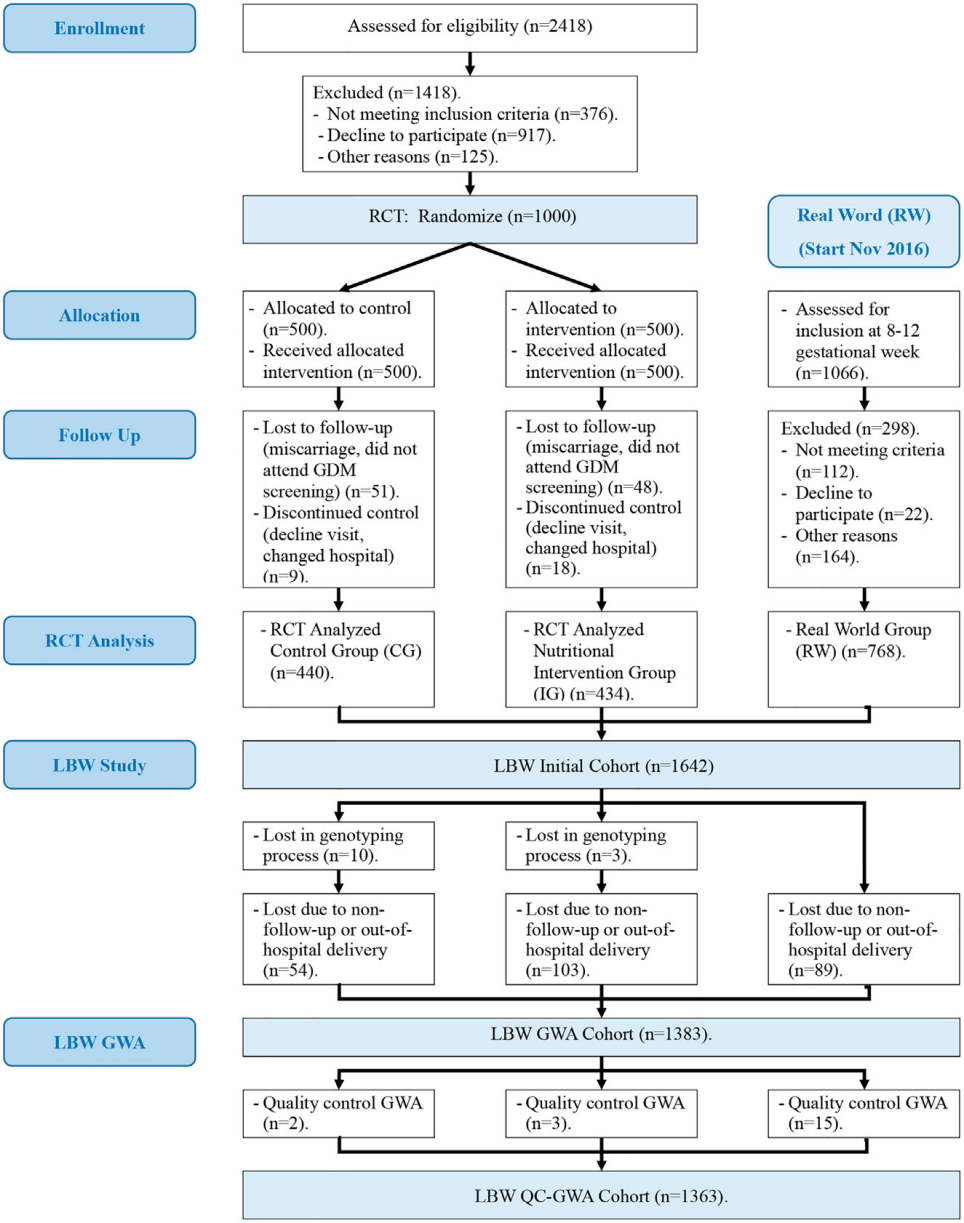
Flow diagram of sample setup

The statistical analysis is based on a cohort of 1,363 women and 86 SNPs who passed all the stages and controls and gave birth to a newborn, of which 72 (5.2%) were LBW (Figure 1). Table 1 includes demographic, anthropometric and clinical characteristics of the sample of pregnant women. It shows significant differences in the distribution of the number of newborns regarding LBW status when adjusted by ETHN (*p* = 0.04), GDM (*p* = 0.01), and NI (*p* = 0.04) variables. Likewise, among the maternal anthropometric and metabolic characteristics considered, only WEIGHT (*p* = 0.01) significantly influenced LBW.

**Table 1:** Main characteristics of pregnant women included in the study

		Low birth weight			
		Controls	Cases	*p* value	Test
Ethnicity	Caucasian	849_a_	57_b_		
		93.7%	6.3%		
	Hispanic	406_a_	15_a_		
		96.4%	3.6%		
	Other	36_a_	0^1^		
		100.0%	0.0%	0.04	Fisher-Freeman-Halton Exact Test
Gestational diabetes mellitus	NO	1080_a_	51_b_		
		95.5%	4.5%		
	YES	211_a_	21_b_		
		90.9%	9.1%	0.01	Fisher-Freeman-Halton Exact Test
Nutritional intervention	Control group	349_a_	25_a_		
		93.3%	6.7%		
	Intervention group	316_a_	9_b_		
		97.2%	2.8%		
	Real world group	626_a_	38_a_		
		94.3%	5.7%	0.04	Fisher-Freeman-Halton Exact Test

		*n*-count	Controls	Cases	*p* value	Test
Maternal	Age	1,363	33.00 [30.00–36.00] years	34.50 [30.00–37.00] years	0.20	Mann-Whitney U
	Height	1,361	1.63 [1.58–1.67] m	1.62 [1.57–1.65] m	0.10	Mann-Whitney U
	Weight	1,353	58.50 [53.50–65.00] kg	56.25 [52.00–61.50] kg	0.01	Mann-Whitney U
	Body mass index	1,351	22.92 [21.08–25.09] kg/m^2^	22.47 [20.72–24.29] kg/m^2^	0.18	Mann-Whitney U
	Glucose	1,361	80.00 [76.00–85.00] mg/dL	81.00 [77.00–87.00] mg/dL	0.25	Mann-Whitney U
	Insulin	1,347	9.10 [5.00–21.80] U/mL	8.70 [4.85–17.25] U/mL	0.87	Mann-Whitney U

Categorical data are presented as absolute and/or relative frequencies.

Numerical data are presented as median and interquartile range [Q1–Q3].

Values in the same row and sub table not sharing the same subscript are significantly different at *p* < 0.05 in the two-sided test of equality for column proportions.

Tests assume equal variances.

Tests are adjusted for all pairwise comparisons within a row of each innermost sub table using the Benjamini-Hochberg correction.

1 This category is not used in comparisons because its column proportion is equal to zero.

### Logistic regression

Association between SNPs and LBW phenotype was evaluated using binary logistic regression models. The base category was the sample minor allele (A1), meaning that it can be a risk allele when OR >1 or a protective allele when OR <1.

Specifically, we used the following genetic inheritance models and corresponding tests: additive, test ADD; dominant, test DOM; recessive, test REC; hetonly, test HET. Several logistic regression model variations were contemplated.

Our initial approach is to pose the most general situation, that is, to analyze the direct relationship between each SNP and LBW. The results of this analysis are valid in themselves at the population level and are of interest to increase general knowledge about the genetic influence on LBW.

The next step is to analyze whether the SNPs-LBW relationships can be modulated by the influence of certain maternal characteristics such as age, height, weight, BMI, glucose and insulin levels of the pregnant woman, data that are systematically recorded in the pregnancy follow-up. Therefore, we consider the SNPs-LBW association models adjusted for each of following numerical variables: maternal age (AGE), height (HEIGHT), weight (WEIGHT), body mass index (BMI), glucose (GLUC), and insulin (INSU) at the time of the OGTT. Furthermore, in genome wide association (GWA) it is common to consider the subject’s ethnicity as a possible source of variation, which leads us to consider the SNPs-LBW association models adjusted by ethnicity (ETHN). As indicated previously, the study is part of a more general project, aimed at the control of gestational diabetes (GDM) in a context of NI based on DietMed. This leads us to consider the models adjusted for GDM and NI group.

To consider the eventual unidentified underlying stratification in the data warehouse, according to literature practical recommendations,^31^ we also consider models that include principal components (PC) as adjust variables. Due to low level of incidence of phenotype, which means a small number of cases in the sample, we included only the first principal component PC1, remaining in sample a case counts greater than 10× predictor count for phenotype. Consequently, we reiterated the previous models and tests, adding in each of them the first PC as an additional variable. Finally, we also calculated models and tests that included the interaction between SNP and the adjust variables, so that for each variable indicated above, we repeated models and tests including additionally the interaction term.

In summary, for each SNP we proposed 29 logistic regression models, and we calculated four tests of association with LBW per model, according to genetic inheritance (ADD, DOM, REC, and HET). This means that we have formulated a total of 116 null hypotheses of no association between LBW and each SNP. Table 2 outlines the variables included in each model.

**Table 2:** Binary logistic regression models for association between LBW and SNP

	MODELS																												
*Variables*	M-1	M-2	M-3	M-4	M-5	M-6	M-7	M-8	M-9	M-10	M-11	M-12	M-13	M-14	M-15	M-16	M-17	M-18	M-19	M-20	M-21	M-22	M-23	M-24	M-25	M-26	M-27	M-28	M-29
*SNP*	YES	YES	YES	YES	YES	YES	YES	YES	YES	YES	YES	YES	YES	YES	YES	YES	YES	YES	YES	YES	YES	YES	YES	YES	YES	YES	YES	YES	YES
*ETHNICITY*		YES										YES									YES								
*GDM*			YES										YES									YES							
*NUTRITION INT.*				YES										YES									YES						
*AGE*					YES										YES									YES					
*HEIGHT*						YES										YES									YES				
*WEIGHT*							YES										YES									YES			
*BMI*								YES										YES									YES		
*GLUCOSE*									YES										YES									YES	
*INSULIN*										YES										YES									YES
*PC1*											YES	YES	YES	YES	YES	YES	YES	YES	YES	YES									
*INTERACTION*																					YES	YES	YES	YES	YES	YES	YES	YES	YES

Effects adjusted by maternal features, principal component 1, and interaction (SNP × feature).

M = Model.

YES: Variable included in the model.

To decide if each of the null hypotheses was truly null or, alternatively, could pinpoint an association signal, that is, a discovery, we used the following approach. For each model, we obtained the corresponding tests *p* values using PLINK software. As false discovery rate (FDR) control, we used the *qvalue* package (version 2.34.0) of R software (version 4.3.3), with smoother method option and adjustment of lambda parameter in the interval 0.01–0.95 with increment of 0.01.^32^^,^^33^^,^^34^ This software estimates the overall proportion π_i0_ of true null hypotheses in each model and computes the *q*-values, i.e., minimum FDR incurred when calling discovery a test that has a *p* value equal to or less than the *p* value associated with the *q*-value; also computes the *lfdr*-values (*local false discovery rate)*, i.e., the empirical Bayesian posterior probability that the null hypothesis is true, conditional on the observed *p* value. We rate a test as discovery when *p* value ≤0.05 or *q*-value ≤0.05 or *lfdr* ≤ 0.1, so that each test of each model received a rating of 0, 1, 2, or 3 as an intensity discovery score. For each variant, we obtained the variant score in a particular logistic regression model by adding the scores of the four genetic tests in the model, so its value is between 0 and 12. In addition, the total score of a variant is obtained as the sum of the scores in all logistic regression models, so its value is between 0 and 348. In the comparative analyses, score values have been rescaled to the corresponding percentual scale.

Table S1 shows the numerical characteristics of variants. Table S2 shows the results of each of the four genetic inheritance tests calculated for each of the 29 models of binary logistic regression proposed, Tables S2A to S2.AC, respectively. Table S3 is a reduced version of Table S2, including only the variants that show a discovery signal in any test of the corresponding model. Table S4 presents scores of variants that have obtained a discovery signal at least in one of the proposed tests. Table S5 shows the scores of discoveries by logistic regression models, genetic models, and the total score.

Table 3 includes only the SNPs with strictly positive total score, along with the corresponding logistic regression results, in the following two relevant cases: (1) model and test for which the SNP reaches the *lowest* OR among the tests in which it has been classified as discovery, that is, the lower-risk/higher-protection situation; (2) model and test for which the SNP reaches the *highest* OR among the tests in which it has been classified as discovery, that is, the highest-risk/lowest-protection situation.

**Table 3:** Low birth weight and SNP association

	
						Minimum Risk/Maximal Protection								Maximum Risk/Minimal Protection							
CHROM	LOCUS	ID	A1	A1_FREQ	OBS_CT	MODEL	TEST	OR	L95	U95	*p*-value	q-value	lfdr	MODEL	TEST	OR	L95	U95	*p*-value	q-value	lfdr
1	MTHFR	rs1801131	G	0.2470	1342	SNP_HEIGHT	ADD	1.37	0.93	2.02	0.1091	0.0422	0.0827	SNP_HEIGHT	HET	1.77	1.09	2.89	0.0213	0.0488	0.0979
1	LYPLAL1	rs2785980	C	0.4033	1360	SNP_HEIGHT	REC	0.21	0.07	0.57	0.0026	0.1027	0.1447	SNP_GDM_PC1	ADD	0.57	0.38	0.85	0.0059	0.0078	0.0139
2	FIGN	rs2119289	C	0.1198	1361	SNP_HEIGHT_PC1	HET	0.55	0.28	1.08	0.0835	0.0391	0.5207	SNP_GDM_PC1	DOM	0.58	0.30	1.12	0.1031	0.0273	0.2079
2	COBLL1	rs7607980	C	0.1215	1362	SNP_HEIGHT	ADD	1.49	0.93	2.39	0.0980	0.0391	0.0812	SNP_HEIGHT	REC	4.28	1.20	15.35	0.0255	0.1272	0.1640
2	G6PC2	rs560887	T	0.2135	1363	SNP_HEIGHT_PC1	DOM	0.60	0.35	1.02	0.0586	0.0209	0.2613	SNP_WEIGHT_PC1	ADD	0.67	0.43	1.05	0.0785	0.0455	0.3249
2	NYAP2	rs2943634	A	0.2744	1350	SNP_WEIGHT_PC1	HET	0.64	0.38	1.07	0.0881	0.0403	0.3083	SNP_HEIGHT_PC1	DOM	0.68	0.41	1.11	0.1248	0.0418	0.5564
2	IRS1	rs1801278	T	0.0745	1362	SNP_GDM_PC1	DOM	1.49	0.82	2.71	0.1930	0.0488	0.4638	SNP_BMI	DOM	1.71	0.95	3.10	0.0760	0.0437	0.0766
3	PPARG	rs17036328	C	0.1126	1363	SNP_GDM_INT	ADD	0.42	0.18	0.98	0.0457	0.1073	0.2598	SNP_GDM_INT	ADD	0.42	0.18	0.98	0.0457	0.1073	0.2598
3	AMT	rs11715915	T	0.2646	1357	SNP_GDM_PC1	ADD	1.38	0.96	1.97	0.0813	0.0417	0.2684	SNP_HEIGHT_INT	DOM	2.02	1.22	3.34	0.0065	0.1959	0.2391
3	IGF2BP2	rs4402960	T	0.2975	1363	SNP_HEIGHT	HET	1.51	0.94	2.44	0.0890	0.0488	0.0979	SNP_HEIGHT	HET	1.51	0.94	2.44	0.0890	0.0488	0.0979
5	IRX1	rs17727202	C	0.0616	1363	SNP_GDM_PC1	DOM	1.67	0.90	3.08	0.1035	0.0273	0.2090	SNP_HEIGHT_INT	HET	1.94	1.06	3.55	0.0317	0.3789	0.3475
5	PCSK1	rs17085593	G	0.2656	1363	SNP_HEIGHT	ADD	1.38	0.94	2.01	0.0973	0.0391	0.0812	SNP_GDM_INT	HET	2.30	1.38	3.83	0.0015	0.0297	0.0333
5	PCSK1	rs6235	G	0.2401	1362	SNP_HEIGHT	ADD	1.38	0.93	2.04	0.1058	0.0414	0.0812	SNP_GDM_INT	HET	2.03	1.22	3.37	0.0061	0.0378	0.0793
6	RREB1	rs9379084	A	0.1207	1363	SNP_HEIGHT_PC1	DOM	0.61	0.33	1.14	0.1197	0.0407	0.5388	SNP_GDM_PC1	DOM	0.61	0.33	1.15	0.1249	0.0322	0.2752
7	GCK	rs1799884	T	0.1935	1362	SNP_BMI_PC1	HET	0.47	0.26	0.88	0.0172	0.0212	0.0472	SNP_HEIGHT	ADD	0.64	0.39	1.04	0.0745	0.0386	0.0812
7	GRB10	rs6943153	T	0.3474	1353	SNP_HEIGHT	ADD	0.71	0.49	1.02	0.0652	0.0386	0.0812	SNP_HEIGHT	ADD	0.71	0.49	1.02	0.0652	0.0386	0.0812
8	ANK1	rs12549902	G	0.4514	1357	SNP_GDM_PC1	ADD	1.38	0.98	1.95	0.0689	0.0359	0.2105	SNP_BMI_INT	REC	1.73	1.00	2.99	0.0489	0.3462	0.3800
8	SLC30A8	rs11558471	G	0.2698	1362	SNP_HEIGHT_PC1	DOM	1.53	0.94	2.49	0.0876	0.0305	0.4075	SNP_GLUC	HET	1.70	1.06	2.74	0.0292	0.1247	0.3315
9	SARDH	rs573904	T	0.2645	1361	SNP_HEIGHT	ADD	1.37	0.95	1.96	0.0920	0.0386	0.0812	SNP_HEIGHT	ADD	1.37	0.95	1.96	0.0920	0.0386	0.0812
10	CDC123	rs11257655	T	0.2399	1363	SNP_INSU_INT	DOM	0.50	0.29	0.87	0.0148	0.6405	0.9590	SNP_HEIGHT_PC1	ADD	0.63	0.40	1.00	0.0491	0.0275	0.3289
10	CDC123	rs12779790	G	0.1773	1362	SNP_AGE_INT	DOM	0.52	0.28	0.98	0.0426	0.4281	0.4055	SNP_HEIGHT_PC1	DOM	0.62	0.36	1.10	0.1004	0.0347	0.4642
10	HHEX	rs7923866	T	0.4060	1362	SNP_WEIGHT_INT	HET	0.56	0.32	0.99	0.0457	0.1435	0.3395	SNP_WEIGHT_INT	HET	0.56	0.32	0.99	0.0457	0.1435	0.3395
10	TCF7L2	rs4506565	T	0.3151	1363	SNP_BMI_INT	ADD	1.44	1.01	2.05	0.0458	0.2079	0.2422	SNP_HEIGHT	DOM	1.97	1.19	3.28	0.0089	0.0554	0.1006
11	CRY2	rs11605924	C	0.4949	1363	SNP_INSU_INT	REC	0.13	0.03	0.71	0.0179	0.5395	0.9233	SNP_INSU_INT	HET	1.72	1.05	2.82	0.0322	0.8612	0.9455
12	OASL	rs7957197	A	0.1746	1360	SNP_HEIGHT	ADD	1.51	1.02	2.25	0.0405	0.0386	0.0812	SNP_HEIGHT	REC	3.40	1.46	7.90	0.0045	0.1272	0.1540
12	FBRSL1	rs10747083	G	0.3190	1362	SNP_GDM_PC1	REC	0.21	0.05	0.85	0.0294	0.0207	0.0894	SNP_GDM	HET	1.49	0.92	2.40	0.1046	0.0496	0.3234
15	C2CD4B	rs11071657	G	0.4493	1362	SNP_AGE_PC1	ADD	1.54	1.07	2.22	0.0190	0.0508	0.0764	SNP_GLUC_PC1	DOM	1.88	1.06	3.34	0.0308	0.0323	0.0830
16	FTO	rs8050136	A	0.3433	1359	SNP_HEIGHT	ADD	1.38	0.97	1.94	0.0704	0.0386	0.0812	SNP_HEIGHT	DOM	1.72	1.03	2.88	0.0381	0.0554	0.1006
17	GLP2R	rs17676067	C	0.2215	1361	SNP_GDM_PC1	HET	1.48	0.91	2.42	0.1164	0.0499	0.4011	SNP_ETHN_INT	REC	3.68	1.58	8.54	0.0024	0.2691	0.5273
19	PEPD	rs731839	G	0.3828	1357	SNP_HEIGHT	ADD	1.42	1.00	2.01	0.0495	0.0386	0.0812	SNP_GDM_PC1	REC	2.36	1.32	4.22	0.0037	0.0101	0.0141
20	TOP1	rs6072275	A	0.1417	1362	SNP_GDM_PC1	DOM	1.48	0.89	2.46	0.1289	0.0329	0.2878	SNP_HEIGHT_INT	DOM	1.68	1.00	2.82	0.0496	0.3274	0.3809
20	ZHX3	rs17265513	C	0.1495	1361	SNP_GDM_PC1	HET	1.55	0.93	2.59	0.0937	0.0410	0.2917	SNP_HEIGHT	REC	2.96	1.12	7.86	0.0291	0.1272	0.1680
20	SLC17A9	rs3746750	A	0.3341	1359	SNP_ETHN_PC1	ADD	1.50	1.05	2.16	0.0261	0.0902	0.1694	SNP_GDM_INT	DOM	2.47	1.38	4.43	0.0024	0.0340	0.0570

Model and Test that show minimum/maximum risk (maximal/minimal protection) for discoveries variants.

CHROM, Chromosome code; LOCUS, Locus/Gene; ID, Variant ID; A1, Counted allele in logistic regression; A1_FREQ, A1 allele frequency; OBS_CT, Number of samples in regression; OR Odds Ratio; L95-U95, OR Lower-Upper Confidence Bounds (95%); q-value, minimum False Discovery Rate; lfdr, local false discovery rate.

**Figure 2:**
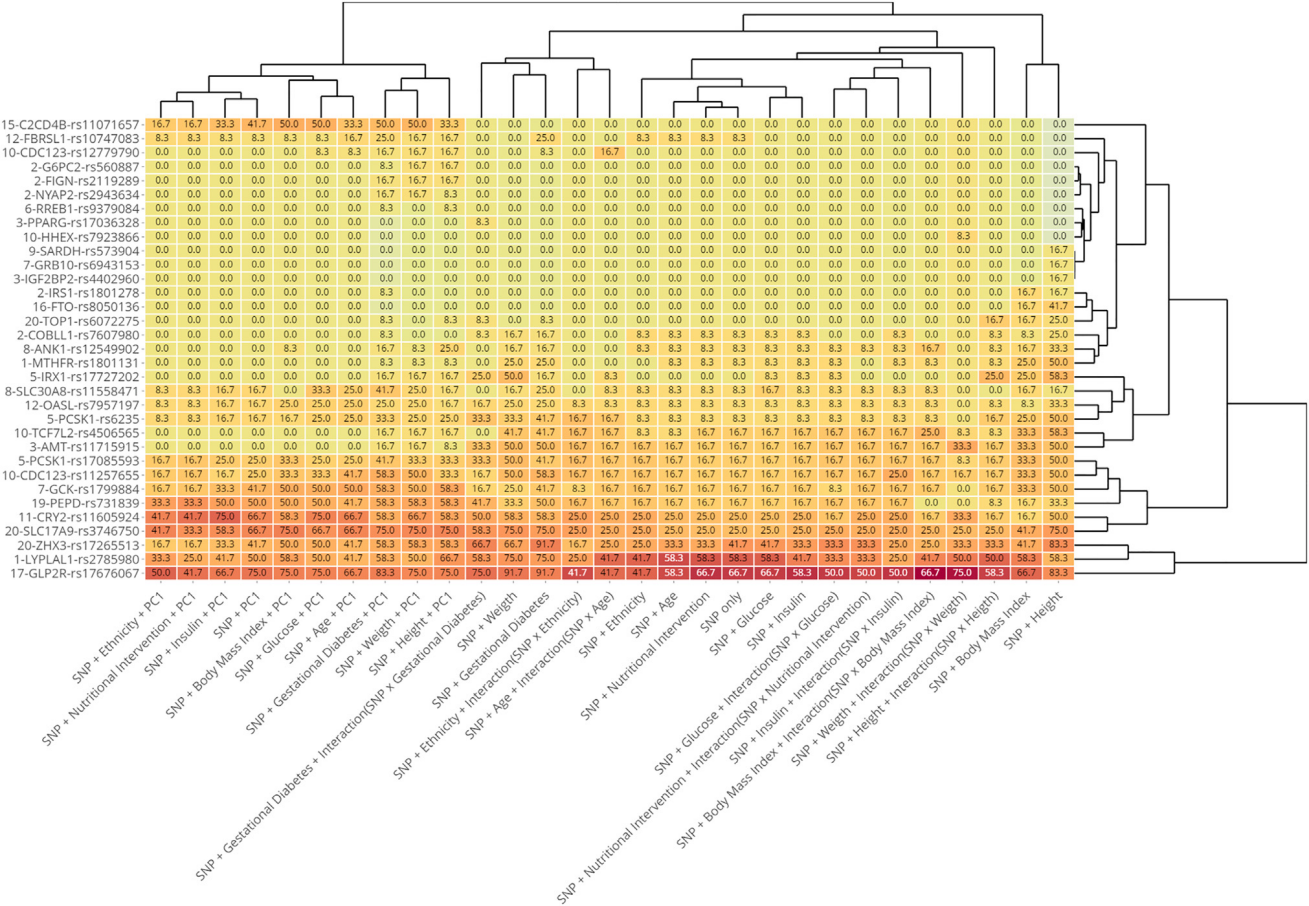
Heatmap and dendrograms derived from LBW-SNP association based on percentage scores that a discovery has achieved in logistic regression models

Figure 2 shows the model scores heatmap of the LBW-SNP association as well as the proximity dendrograms between variants and between models, derived from variants with positive total score. We use the metric that results from rescaling the total score to a percentage scale from 0 to 100.

### Bioinformatics analysis and gene enrichment

We reduced the set of SNPs to those for which any of the proposed association tests received a discovery rating strictly greater than zero. With the corresponding set of proteins/genes as initial data, we performed a functional enrichment process using String 12 software https://string-db.org.^35^ We selected the following options: full string network, edges mean evidence, all active interaction sources, and minimum required interaction score equal to 0.15. The results included the interaction network between proteins/genes resulting from the LBW discoveries, the enrichment terms of these proteins/genes, and information related to the annotations available in String 12 on proteins/genes.

**Figure 3:**
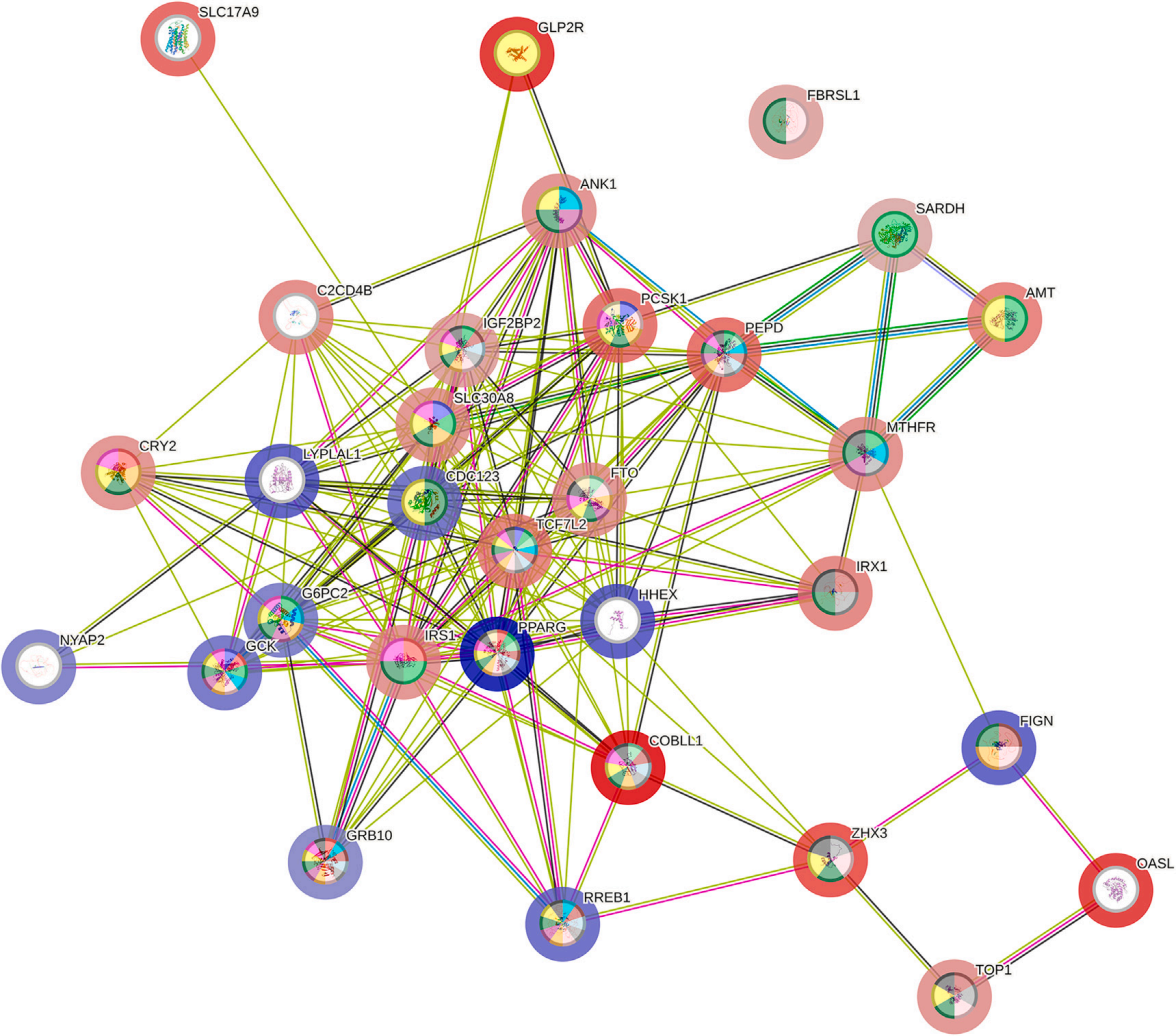
Network of interactions between proteins/genes identified by mapping of low-birth-weight GWA discoveries Nodes’ inner color represent the SNP association with terms in Table 4, halo color represents the protective (blue) or risk (red) nature of the SNP, with the intensity being proportional to the corresponding OR in Table 3, and colors of the edges represent the active interaction sources.

Figure 3 shows the String 12 interaction network between proteins/genes resulting from discoveries mapping. The network permanent link is https://version-12-0.string-db.org/cgi/network?networkId=b0y0rXL0NYIR.

Network statistics are: 31 nodes, 167 edges, average node degree 10.8, expected number of edges: 40, protein-protein-interaction enrichment *p* value <1.0E-16.

Table S6 summarizes the gene enrichment process executed by String 12. Specifically, Table S6A shows the description of the genes/proteins, Table S6B includes various interaction scores between genes/proteins, and Table S6C shows the list of 126 enriched terms in the following 5 categories: GO Process, 19 terms; Search Tool for the Retrieval of Interacting Genes/Proteins (STRING) clusters, 2 terms; Monarch, 91 terms; Diseases, 8 terms; and UniProt Keywords, 2 terms. Figure S1 presents a Manhattan Plot graphic with the strength and fdr terms from Table S6C.

**Figure 4:**
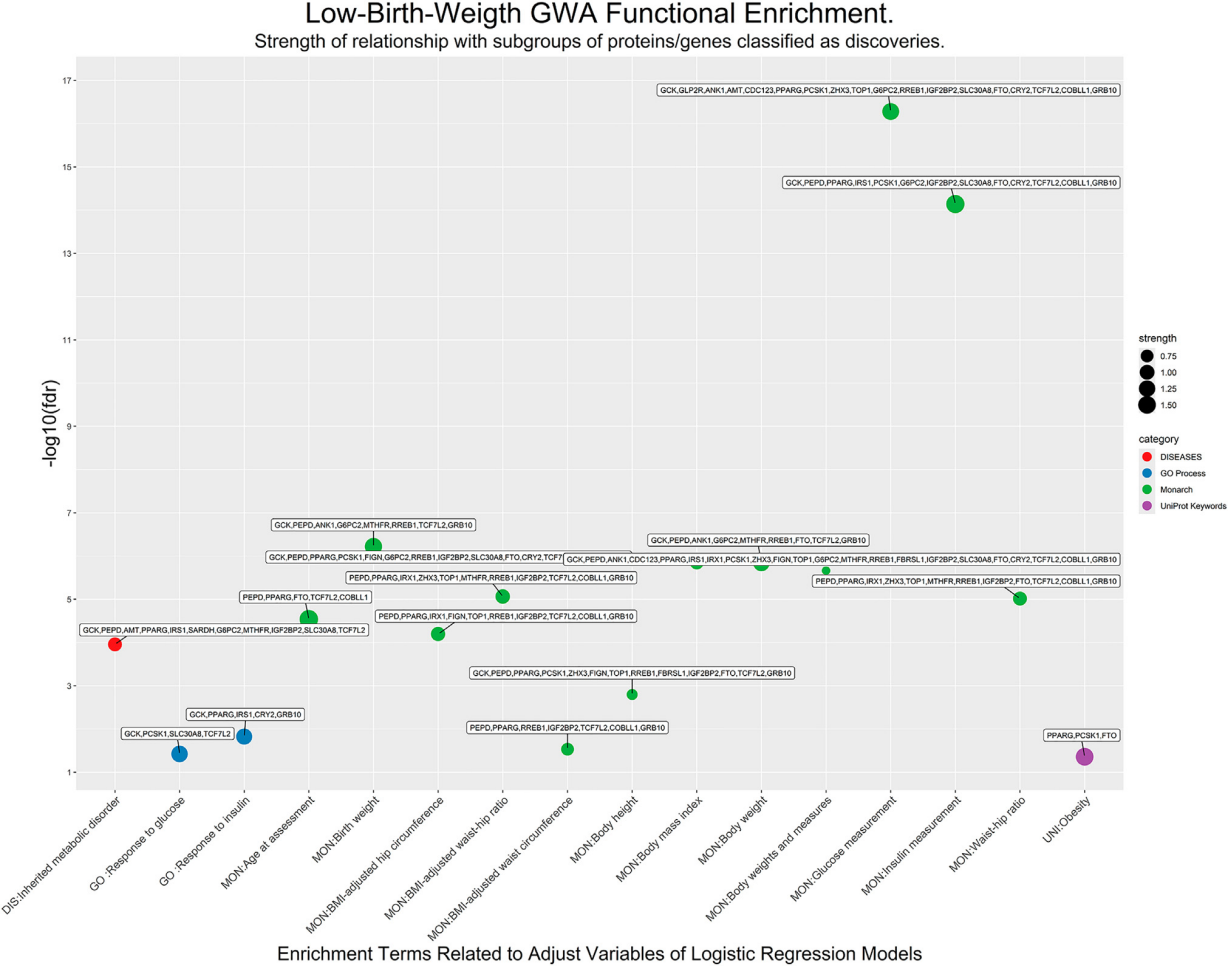
Low-birth-weight GWA functional enrichment x axis includes functional enrichment terms directly related to model variables considered in the GWA-LBW study (Table 2). Points refer to proximity clusters between discoveries and the size of the point indicates the strength of the association (Table 4).

Table 4 is a reduced version of Table S6C obtained by manually extracting main terms specifically related to methodological framework features, and Figure 4 shows the corresponding Manhattan-plot, including the strength and fdr of each enrichment term and the subset of variants associated with each of them.

**Table 4:** Functional enrichment terms linked to low-birth-weight directly related to model variables considered in the GWA-LBW study

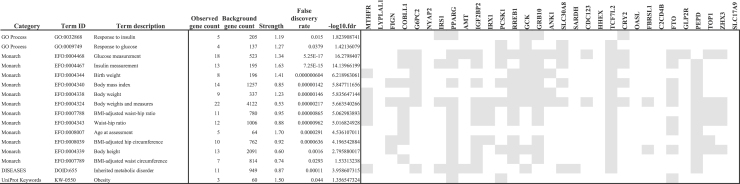

### Main general findings

Our study begins with 110 variants, of which 86 (78.2%) passed rigorous quality control and configured a set of SNPs in approximate linkage equilibrium. 33 variants, 38.4% of analyzed variants, reached a positive discovery rate.

The association LBW-SNPs shows coherent patterns both in the logistic regression models and individually for each SNP. In the three methodological groups of models on which our study is based, (SNP+Variable, SNP+Variable+PC1, and SNP+Variable+Interaction), the association signals are more intense when the adjust variables are HEIGHT, GDM, and WEIGHT, it is slightly lower when adjusted by BMI, and reaches a level similar to model base, only SNP effect, in the models adjusted by ETHN, NI, AGE, GLUC, and INSU. In general, the association signals increase with respect to base models when principal component variable PC1 is introduced and decreases when interaction term is considered.

In the simplest model, SNP only, a positive rate was observed in 18 variants (Table S2A). The most notable discoveries are LYPLAL1/rs2785980/C, protective, min OR = 0.31 (REC), max OR = 0.51 (ADD); GLP2R/rs17676067/C, risk, min OR = 2.02 (ADD), max OR = 2.99 (REC); ZHX3/rs17265513/C, risk, min OR = 1.71 (HET), max OR = 2.84 (REC), and SLC17A9/rs3746750/A, risk, min OR = 1.55 (ADD), max OR = 2.17 (DOM).

When considering all logistic regression models, we can classify the discoveries into four groups defined by the quartiles Q1–Q4 of the percentual discovery score (pds).

(1) *Discoveries in Q1* (pds ≥ 26.7%). Risk variants: GLP2R/rs17676067/C, PEPD/rs731839/G, ZHX3/rs17265513/C, SLC17A9/rs3746750/A. Protector variants: LYPLAL1/rs2785980/C, GCK/rs1799884/T, CDC123/rs11257655/T. Risk/protector variant depending on genetic inheritance test: CRY2/rs11605924/C. These variants have reached statistical significance in practically all proposed logistic regression models.
(2) *Discoveries in Q2* (9.8% ≤ pds < 26.7%). Risk variants: AMT/rs11715915/T, IRX1/rs17727202/C, PCSK1/rs6235/G, PCSK1/rs17085593/G, SLC30A8/rs11558471/G, TCF7L2/rs4506565/T, OASL/rs7957197/A, C2CD4B/rs11071657/G. Most of the variants in this quartile reach statistical significance in models without PC1, but it is noteworthy that C2CD4B variant only reaches positive rates in models in which the principal component PC1 appears.
(3) *Discoveries in Q3* (1.7% ≤ pds < 9.8%). Risk variants: COBLL1/rs7607980/C, FTO/rs8050136/A, TOP1/rs6072275/A, MTHFR/rs1801131/G, ANK1/rs12549902/G. Protector variants: FIGN/rs2119289/C, CDC123/rs12779790/G, FBRSL1/rs10747083/G.
(4) *Discoveries in Q4* (pds < 1.7%). Risk variants: IRS1/rs1801278/T, IGF2BP2/rs4402960/T, SARDH/rs573904/T. Protector variants: G6PC2/rs560887/T, NYAP2/rs2943634/A, PPARG/rs17036328/C, RREB1/rs9379084/A, GRB10/rs6943153/T, HHEX/rs7923866/T.

Nodes’ halo color in Figure 3 indicates the main character of variant, blue for protection and red for risk, while color intensity is proportional to the OR of logistic regression model with greater risk/less protection, Table 3.

Table 4 and Figure 4 include the 16 enrichment terms extracted from Table S6C obtained from the GWA discoveries, for their specific relationship with methodological framework features. Terms with greatest strength are the following: *Age at assessment:* strength = 1.70, fdr = 2.91E-05; *Insulin measurement:* strength = 1.63, fdr = 7.25E-15; *Obesity:* strength = 1.50, fdr = 0.044; *Birth weight:* strength = 1.41, fdr = 7.04E-07; *Glucose measurement:* strength = 1.34, fdr = 5.25E-17.

Colors inside the nodes in Figure 3 indicate the relationship of the protein/gene with the corresponding enrichment terms included in Table 4.

## Discussion

This work presents an extensive evaluation of the link between the LBW phenotype, as defined by the WHO, with a set of 86 maternal genetic variants. The population forms part of a large healthcare center in Madrid (Spain), which follows-up pregnant women from different ethnographic origins and their offspring, who have undergone a NI developed in a first phase as an RCT and, subsequently, generalized to the entire population. The analysis methodology follows a multifactorial approach and includes maternal genetic information, both genotype and inheritance, along with anthropometric and metabolic characteristics of the future mother that are routinely evaluated during pregnancy.

The GWA design follows the general STrengthening the REporting of Genetic Association studies (STREGA) guidelines,^36^ as well as literature recommendations that advise to introduce some sample principal component(s) as control variables of association models,^31^ to formulate different genetic inheritance tests,^37^ and to perform *fdr* control by means of *qvalue* method.^32^^,^^33^^,^^34^ GWA identified 33 variants that show signals of association with LBW. The searching strategy was based on 29 logistic regression models in each of which four models of genetic inheritance were evaluated.

Figure 2 shows that association patterns are remarkably similar in logistic regression models, and these form three main cluster roughly corresponding to the three approaches used to models configuration: SNP + control variable, adding PC1, and adding interaction. In general, association evidence increases when PC1 is included as adjust variable and decreases when the interaction is considered in models.

The basic model, SNP only, presents 18 discoveries, all of which are in Q1 and Q2 quartiles of score, except MTHFR, COBLL1, and FBRSL1 that are in Q3 quartile.

With some differences, the association pattern observed in SNP only model is repeated in models that included an adjust factor or biometric variable.

Most models with PC1 modify the pattern of basic model. In general, variants that remain in models with PC1 increase the association evidence.

Models that include the interaction term show an uneven pattern. We can point out that interaction model with GDM maintains an association pattern in the variants of the Q1 quartile, while interaction model with WEIGHT maintains a different pattern. The performance of models with interaction is that association evidence decreases, so we understand that its predictive usefulness is weaker and, in any case, deserves further investigation.

The previous discussion allows us to establish that the association patterns obtained in different models of GWA are coherent, and clearly determine the association of SNPs classified as discoveries with the LBW phenotype.

Functional enrichment reveals biological mechanisms underlying the association of LBW and the identified discoveries. Table 4 and Figure 4 show that PCSK1, GCK, GRB10, and CRY2 are associated with the process *response to glucose,* while g*lucose measurement* is associated, in addition to the previous ones, with COBLL1, AMT, IGF2BP2, PCSK1, RREB1, GRB10, ANK1, SLC30A8, SARDH, CDC123, TCF7L2, FTO, GLP2R, TOP1, ZHX3. Moreover, IRS1, PPARG, GCK, GRB10, and CRY2 are associated with *response to insulin* process, while, in addition, COBLL1, G6PC2, IRS1, IGF2BP2, SLC30A8, TCF7L2, FTO, GLP2R, TOP1 and ZHX3 are associated with *insulin measurement.* These facts reinforce the genetic influence of insulin on LBW, reaffirming the role regulator of insulin in fetal growth,^7^ and pointing toward the hypothesis of fetal insulin, which proposes that LBW and T2D in adulthood may be two phenotypes of the same genotype.^38^^,^^39^

In Table 4, the term *birth weight* is associated with MTHFR, G6PC2, RREB1, GCK, GRB10, ANK1, and PEPD, while in the case of the term *body weight*, FTO is added to the previous list. These variants reach values of different ranges in our GWA scores, but we understand that the results allow us to reaffirm the association of these SNPs with LBW.

It can be observed that Table 4 includes protein/genes associated with a relevant number of enrichment terms, such as PPARG, IGF2BP2, RREB1, GRB10, that they have barely obtained any association with LBW in our study.

In this regard, we can point out that variants of PPARG, IGF2BP2, and GRB10 have been associated with weight-related phenotypes, Table 5. Nevertheless, we have not located any reference to association of any of them with LBW. However, RREB1 is associated with offspring birth weight.^21^ These facts suggest that, in our GWA approach, a low discovery score does not necessarily imply the absence of association between the variant and the phenotype. Rather, as corresponds to an exploratory study, the results point the way for a more in-depth study of these variants.

**Table 5:** Variants associated with a relevant number of enrichment terms in Table 4 and, however, present low scores in the logistic regression models with LBW

CHROM	POS	ID	GENE	SYMBOL	A1	DESCRIPTION	ASSOCIATION	REFERENCES	OBSERVATIONS
3	12348985	rs17036328	ENSG00000132170	PPARG	T	Peroxisome proliferator activated receptor gamma.	Fasting blood insulin measurement. Body mass index.	Black et al.^40^	
3	185793899	rs4402960	ENSG00000073792	IGF2BP2	G	Insulin like growth factor 2 mRNA binding protein 2.	Peak insulin response measurement. Type II diabetes mellitus.	Ramos-LeviRodríguez et al.^30^ and Arnoriaga-Rodríguez et al.^41^	
6	7231610	rs9379084	ENSG00000124782	RREB1	A	Ras responsive element binding protein 1.	Appendicular lean mass. Birth weight. Diabetes mellitus. Fasting blood glucose measurement. Heel bone mineral density. Body height. HbA1c measurement. BMI-adjusted hip circumference. Body mass index. Parental genotype effect measurement. Type II diabetes mellitus.	Warrington et al.^21^	
7	50723882	rs6943153	ENSG00000106070	GRB10	T	Growth factor receptor bound protein 10.	Fasting blood glucose measurement.	Holt and Siddle^42^	Described as enigmatic regulator of insulin action.

CHROM, Chromosome code; POS, Base-pair coordinate [GRCh38]; ID, Variant ID; A1, Counted allele in logistic regression.

On the contrary, several protein/genes, such as GLP2R, SLC17A9, OASL, C2CD4B, and LYPLAL1, barely show association with terms in Table 4. However, in our study, they present high association scores with LBW. Table 6 shows some characteristics of these discoveries.^43^^,^^44^

**Table 6:** Variants with high scores in the logistic regression models with LBW and, however, are associated with a low number of enrichment terms in Table 4

CHROM	POS	ID	GENE	SYMBOL	A1	DESCRIPTION	ASSOCIATION	Reference	OBSERVATIONS
1	219527177	rs2785980	ENSP00000355895	LYPLAL1	C	Lys phospholipase-like protein 1	Phenotypes: adiponectin measurement, fasting blood insulin, waist-hip ratio.	Spracklen et al.^45^, Wheeler et al.^46^ and Heid et al.^47^	
12	121022883	rs7957197	ENSG00000135114	OASL	A	2′-5′-oligoadenylate synthetase like. Belongs to the 2-5A synthase family.	Biological process: immune system process, response to virus. Phenotypes: C-reactive protein levels, cardiovascular disease risk factors, hematocrit, hemoglobin, inborn genetic diseases, N-glycan levels, inflammation.	Ding et al.^19^	Variant rs7957197 has been mentioned in various publications in relation to T2D, usually mapped to the HNF1A gene, which is more than 20knt away from the position of the variant in the base-pair coordinate GRCh.38
15	62141763	rs11071657	ENSG00000205502	C2CD4B	G	C2 calcium dependent domain containing 4B. Belongs to the C2CD4 family and may regulate cell architecture and adhesion.	Biological process: regulation of cell adhesion. Phenotypes: fasting blood glucose, fasting blood proinsulin levels, pulse pressure, L-selectin levels, height, inborn genetic diseases.	Dupuis et al.^48^ and Jung et al.^49^	
17	9888058	rs17676067	ENSG00000065325	GLP2R	C	Glucagon Like Peptide 2 Receptor. Is a receptor for glucagon-like peptide 2 mediated by G proteins that activate adenylyl cyclase.	Biological process: positive regulation of cell population proliferation, and cellular response to glucagon, adenylate cyclase-modulating G protein-coupled receptor signaling pathway. Phenotypes: stimulus glucose-dependent insulinotropic polypeptide (GIP) levels in response to oral glucose tolerance test (fasting), type 2 diabetes, GDM, inborn genetic diseases.	Ramos-Levi et al.^30^ and Scott et al.^50^	
20	62967547	rs3746750	ENSG00000101194	SLC17A9	A	Solute carrier family 17 member 9. Involved in vesicular storage and exocytosis of ATP and may accumulate ATP and other nucleotides in secretory vesicles such as adrenal chromaffin granules and synaptic vesicles.	Biological process: ATP and ADP transport. Phenotypes: disseminated superficial actinic porokeratosis, inborn genetic diseases, GDM.	Ramos-Levi et al.^30^	

CHROM, Chromosome code; POS, Base-pair coordinate [GRCh38]; ID, Variant ID; A1, Counted allele in logistic regression.

Our results are in line with the GWA studies of birth weight focused on maternal genetic variants. Our GWA includes some variants that Warrington et al. significantly associate with birth weight,^21^ specifically, the variants G6PC2/rs560887, RREB1/rs9379084, TCF7L2/rs7903146, ADCY5/rs11708067, and GLIS3/rs10814916. Our study agrees on the direction of the association and its statistical significance for G6PC2, RREB1, and TCF7L2. However, our analysis has not given a signal of discovery for GLIS3 and ADCY5. On the other hand, at locus level, our study shares with the GWA of Warrington et al. the following discoveries: MTHFR, GCK, GRB10, and ANK1.^21^

An additional consideration can be drawn from our study. Traditional management of some pregnancy complications, such as GDM, focuses on tight glycemic control to prevent eventual macrosomia. This may cause a bias toward increasing the incidence of LBW.^11^ The results of this work may contribute to preventing this unintended consequence.

### Limitations of the study

The limitations of our study are mainly derived from the general research project, about NI and GDM control, in which it is framed. Analysis of SNPs does not include variants specifically related only to birth weight,^21^ or growth retardation.^4^ On the other hand, as mentioned in the introduction, only maternal genetic information was available, without that of the newborn or the paternal information.

### Conclusion

Paraphrasing the WHO, the proportion of infants with LBW is an indicator of a multifaceted public health problem that includes long-term maternal malnutrition, ill-health, and poor health care during pregnancy.

Our study identifies genetic and epigenetic risk factors that allow the prevention of LBW. These factors include a set of maternal SNPs associated with LBW in the newborn. The association is modulated by several maternal characteristics, which are usually monitored during pregnancy. Our overall analysis proves that the genetic variants lead to proximity clusters, grouped into subsets of variants statistically associated with underlying biological processes or other maternal characteristics, which, on their part, allow early prevention of the eventual risk of LBW.

Our study allows to conclude that the prevention and reduction of the prevalence of LBW must be achieved through attention to issues such as the careful observation of certain maternal phenotypes, as well as the persistent recommendation to pregnant women regarding an appropriate diet and modus vivendi, together with the eventual incorporation of the maternal genome analysis, which leads to more personalized monitoring of pregnancy.

## Resource availability

### Lead contact

Requests for further information and resources should be directed to and will be fulfilled by the lead contact, Ana M. Ramos-Levi (ana_ramoslevi@hotmail.com).

### Materials availability

This study did not generate new unique reagents.

### Data and code availability

• Data that support the findings of this study are available from the corresponding author upon reasonable request.
• This paper does not report original code.
• Any additional information required to reanalyze the data reported in this paper is available from the lead contact upon request.

## Acknowledgments

We wish to acknowledge our deep appreciation to the administrative personnel, nurses, and dieticians from the Laboratory Department (María Victoria Sáez de Parayuelo and Jose Luis Espadas) and the Pregnancy and Diabetes Unit and to all members of the Endocrinology and Nutrition and Obstetrics and Gynecology departments of the San Carlos Clinical Hospital, and to Primary Care Practitioners and Family Doctors in the central area of Madrid.

This research was funded by grants from Fundación para Estudios Endocrino-metabólicos, IdISSC Hospital Clínico San Carlos, from the The Instituto de Salud Carlos III/MICINN of Spain under grant number PI20/01758, and European Regional Development Fund (FEDER) “A way to build Europe.” The design and conduct of the study; collection, management, analysis, and interpretation of the data; preparation, review, and approval of the manuscript; and decision to submit the manuscript for publication are the responsibilities of the authors alone and independent of the funders.

## Author contributions

Conceptualization: A.M.R.-L., R.M.O., M.A.R., P.M.-M., and A.C.-P.; data curation: A.M.R.-L, L.d.V., and A.C.-P.; formal analysis: A.M.R.-L.; methodology: V.M., J.V., L.d.V., A.B., and A.C.-P.; investigation: A.M.R.-L., L.M., M.Z., I.M., C.F., A.D.-P., M.P.d.M., C.M., M.A.-R., A.B., M.J.T., and A.C.-P.; draft preparation: A.M.R.-L. and A.C.-P.; draft review and editing: A.M.R.-L. and A.C.-P.; supervision: P.M.-M. and A.C.-P.; funding acquisition: A.C.-P. All authors confirm that they had full access to all the data in the study, have reviewed the final manuscript, and accept responsibility to submit for publication.

## Declaration of interests

The authors declare no competing interests.

## STAR★Methods

### Key resources table

**Table:** 

REAGENT or RESOURCE	SOURCE	IDENTIFIER
**Biological samples**		

Genomic DNA extracted from EDTA-stabilized blood samples	This paper	

**Deposited data**		

Genome assembly GRCh38	Genome Reference Consortium (GRC)	https://www.ncbi.nlm.nih.gov/datasets/genome/GCF_000001405.26/
Phenotype driven discovery	Monarch Initiative	https://monarchinitiative.org/
The Human Phenotype Ontology (HPO)	Monarch Initiative	https://hpo.jax.org/
Ensembl Variant Effect Predictor (VEP)	Ensembl	https://www.ensembl.org/info/docs/tools/vep/index.html
Raw and analyzed data	This paper	Available from the lead contact upon request (ana_ramoslevi@hotmail.com)

**Oligonucleotides**		

IPLEX MassARRAY PCR and extension primers for each target SNP (Table S3-1)	This paper	

**Software and algorithms**		

IBM SPSS Statistics for Windows, Version 29.0.1.0(171)	Armonk, NY: IBM Corp.	https://www.ibm.com/es-es/products/spss-statistics
R software (version 4.3.3)	R Software Foundation	https://www.r-project.org/
Plink 1.9, PLINK 2.00 Alpha 5.10	Storey et al.^33^	https://www.cog-genomics.org/plink/
qvalue package (version 2.34.0) of R software (version 4.3.3)	Szklarczyk et al.^35^, Little et al.^36^ and Liu et al.^37^	https://www.bioconductor.org/packages/release/bioc/html/qvalue.html
String 12	Hattersley and Tooke^38^	https://string-db.org
ggplot2 3.5.1	Reim et al.^39^	https://ggplot2.tidyverse.org/index.html
ggrepel 0.9.6	Black et al.^40^	https://github.com/slowkow/ggrepel.
heatmaply 1.4.3	Arnoriaga-Rodríguez et al.^41^	https://talgalili.github.io/heatmaply/articles/heatmaply.html

### Experimental model and study participant details

Data included in this study have been configured as summarized below. During the years 2014-16, a total of 2418 women attending their first gestational visit (GW) at 8–12, Visit 0, with FBG <92 mg/dL, were assessed for inclusion in a randomized clinical trial (RCT). They were invited to participate upon their first ultrasound visit, between 12 and 14 GW (Visit 1). Gestational age at entry for inclusion was based on the one obtained in this first ultrasound. The inclusion criteria were: ≥18 years old, single gestation, acceptance of participation in the study, and signature of the consent form. The exclusion criteria were: gestational age at entry >14 GW, intolerance to nuts or extra virgin olive oil (EVOO), and medical conditions or pharmacological therapy that could compromise the effect of the intervention and/or the follow-up program. From this initial group, a sample of 1000 women was selected and randomly divided into two groups of the same size, according to NI. Allocation to control group (CG) and IG was performed by building a stratified randomization with permutated block-randomization, stratified by age (18–29, 30–34 and ≥35), pregestational body mass index (BMI) (<25, 25–29.9 and ≥30 kg.m2), parity (1 or >1), and ethnicity, classified as Caucasian (Spanish and Slavic), Hispanic and other, in an allocation ratio of (1:1) in blocks of 4–6. Due to the nature of the RCT design, participants, staff and the dietician were aware of the allocation assignments. Allocation to groups remained unknown to the statistician and research assistant. Both groups were given the same basic Mediterranean Diet (MedDiet) recommendations: ≥ two servings/day of vegetables, ≥ three servings/day of fruit (avoiding juices), three servings/day of skimmed dairy products, wholegrain cereals, two-three servings of legumes/week, moderate to high consumption of fish; a low consumption of red and processed meat, avoidance of refined grains, processed baked goods, pre-sliced bread, soft drinks and fresh juices, fast foods and precooked meals. They were also recommended to walk ≥30 min/day. These recommendations were given to women by different parties, depending on the group they were allocated to. On one hand, participants allocated to IG received lifestyle guidance from dieticians one week after inclusion in a unique 1-h group session. The key IG recommendation was a daily consumption of at least 40 mL of EVOO and a handful (25-30g) of pistachios. To ensure the consumption of the minimum amount recommended, women were provided at Visit 1 and 2 with 10 L of EVOO and 2 kg of roasted pistachios each. This way, they had available 1L of EVOO and 150g of roasted pistachios weekly, throughout their pregnancy. Women in the CG, however, were advised by midwives to restrict consumption of dietary fat, including EVOO and nuts. These recommendations are provided in local antenatal clinics as part of the available guidelines in pregnancy standard care. The number of visits for the study was alike in both groups. All women were followed-up taking advantage of their scheduled standard-practice laboratory appointments. This was at first ultrasound visit (Visit 1), at 24–28 GW (Visit 2), third trimester evaluation at 36–38 GW (Visit 3) and at delivery. Nutritional guidance was reinforced at each visit for both groups. Dietary recommendations were individualized at each visit, in the context of usual recommendations. These recommendations were given aiming to reduce the caloric content of their diet when gestational weight gain exceeded the goal, by either the dietician (IG) or the midwife (CG). The study was completed by 874 women (440/434, CG/IG). This group is the initial sub-cohort of this paper.

The RCT concluded that an early NI with a supplemented MedDiet reduces the incidence of GDM.^28^ Based on these results, our hospital recommended the adoption of this NI (i.e., MedDiet enriched with EVOO and nuts), without providing these specific products, to all pregnant women, from the beginning of gestation, in real word.^29^ Thus, from November 2016 onwards, every pregnant woman who attended the first gestational visit were invited to participate in our study based on the implementation of the RCT results in clinical practice. In accordance with the inclusion and exclusion criteria indicated above, a new sub-cohort (real-world group, RW) was defined, with 768 samples that are included in this study. Therefore, a total of 1642 pregnant women, with their corresponding fetus, comprised the initial sample of this study, Figure 1.

### Method details

#### Genotyping

Genomic DNA was extracted from EDTA-stabilized blood samples, taken between 08.00 and 09.00 a.m. after an overnight fast, at the time of the OGTT for screening of GDM, between 12 and 14 GW (Visit 1), using the Maxwell RSC instrument (Promega, Dubendorf, Switzerland). Genotyping was performed by IPLEX MassARRAY PCR using the Agena platform (Agena Bioscience, SanDiego, CA). An Agena Bioscience Compact MassArray Spectrometer was used to perform MALDI-TOF mass spectrometry according to the iPLEX Gold Application Guide. The Typer 4 software package (Agena Bioscience) was used to analyze the resulting spectra, and the composition of the target bases was determined from the mass of each extended oligo. These panels were designed in collaboration with PATIA BIOPHARMA S.A. (www.patiadiabetes.com) and genotyping was performed at the Agena platform located at the Epigenetics and Genotyping laboratory, Central Unit for Research in Medicine (UCIM), Faculty of Medicine, University of Valencia, Valencia, Spain. More details can be found at Ramos-Leví.^30^

Thirteen samples were lost in the genotyping process. In addition, 246 pregnant women did not give birth in our hospital, mainly for personal and family reasons. Consequently, the GWA input data included 1383 samples, Figure 1.

#### Single nucleotide polymorphisms list and characteristics

For each SNP, Table S7A includes the references used for selection, the chromosome code, base-pair coordinate GRCh38, variant identification, reference allele, and URL location from dbSNP.^43^ Main characteristics of the variants were extracted from the Ensembl database using Variant Effect Predictor.^44^ Specifically, for each variant, we obtained the annotations of consequences, biotypes, genes, and symbols (Tables S7B–S7E). We used the information collected in Tables S7C–S7E to map each variant to a symbol gene. Variants that show a protein-coding biotype, Table S7C, were mapped to the corresponding most relevant symbol gene collected in Tables S7D and S7E. For some variants, the information in Tables S7C–S7E did not allow us to clearly resolve the desired mapping, so we looked directly at Ensembl database to locate the protein code gene closest to these variants. We show the genes that result in mapping in Table S7F and will be included as additional identification of variants.

#### Genome wide analysis quality control

Starting from the initial set of 110 SNPs, quality control process produces first a pruned subset of variants in approximate linkage equilibrium (independent-pairwise 100kb 1 0.8), which excludes 11 variants from the analysis. Next, we removed SNPs with a high missing genotype data (GENO >5%, 1 variant), removed SNPs due to Hardy-Weinberg exact test (HWE, *p* < 1.0E-06, 8 variants), and removed SNPs due to allele low frequency threshold (MAF <5%, 4 variants). As a result, our data warehouse included 86 SNPs (Table S7G).

Quality control process of sample genotyping (MIND >5%) eliminated 20 samples, so the size of the study cohort reached 1363 samples (Figure 1), with a total genotyping rate in remaining samples equal to 0.996658.

#### Coding of the Low birth weight phenotype

Samples were coded LBW if their birth weight was less than or equal to 2500g and non-LBW otherwise (WHO criteria).

#### Graphic software

For graphic representation, the following R packages we used: *ggplot2* (version 3.5.1),^51^ *ggrepel (*version 0.9.5),^52^ *heatmaply* (version 1.5.0),^53^ and *plotly* (version 4.10.4).^53^

### Quantification and statistical analysis

Statistical analyses regarding patients’ characteristics were performed in IBM SPSS Statistics for Windows, Version 29.0.1.0(171), Armonk, NY: IBM Corp. Categorical data are presented as absolute and/or relative frequencies. The normality of the scale variables will be verified using the Lilliefors Corrected Kolmogorov-Smirnov test. Normal variables are presented as mean ± standard deviation, while the median and interquartile range will be used for non-normal variables. Qualitative characteristics were compared with Fisher-Freeman-Halton Exact Test. Quantitative characteristics were compared with Student’s t test or Mann-Whitney U test, depending on whether their distribution was normal or not. A two-sided *p*-value ≤0.05 was considered statistically significant. Tests assume equal variances. Tests are adjusted for all pairwise comparisons using the Benjamini-Hochberg correction.

Logistic regression models were performed using PLINK v.1.9 and PLINK 2.0 Alpha 5.10 software.^33^ PC variables were calculated using Plink 2.0.^54^ As FDR control, we used the *qvalue* package (version 2.34.0) of R software (version 4.3.3).^32^^,^^33^^,^^34^

### Additional resources

This study forms part of a broader project initiated developed in various phases, including a randomized controlled trial (RCT), registered December 4, 2013 at SRCTN84389045 (https://doi.org/10.1186/ISRCTN84389045), and a real-world study, registered October 11th, 2016 at ISRCTN13389832 (https://doi.org/10.1186/ISRCTN13389832), both with approval by the Clinical Trials Committee of the Hospital Clínico San Carlos, Madrid, Spain (July 17, 2013, CI13/296-E and October 1st, 2016, CI16/442-E, respectively), and compliance with the Declaration of Helsinki.

## Supplemental information

Document S1. Figure S1

Table S1. Numerical characteristics of variants that passed all quality control conditions, related to Star methods, Table 2 and Table 3

Table S2. LBW - SNPs association models, related to Table 2 and Table 3

Table S3. Variants that show a discovery signal in any test of the corresponding model, related to Table 2 and Table 3

Table S4. SNP discoveries test score, related to Table 2 and Table 3

Table S5. Regression model scores, genetic model scores, and total scores for SNP discoveries, related to Table 2 and Table 3

Table S6. LBW GWA protein mapping, protein interactions and protein enrichment provided by STRING 12, related to Figure 3 and Figure 4

Table S7. Initial list of variants and their characteristics, related to Star methods
