# Supplementary material for: Maternal genomic profile, gestational diabetes control, and Mediterranean diet to prevent low birth weight
Source: iScience. 2024 Nov 13;27(12):111376. doi: 10.1016/j.isci.2024.111376 (PMC11648256; doi:10.1016/j.isci.2024.111376)
Supplement: Document S1. Figure S1 [file mmc1.pdf]

iScience, Volume *27*

Supplemental information

Maternal genomic profile, gestational diabetes control, and Mediterranean diet to prevent low birth weight

Ana M. Ramos-Levi, Rocío Martín O'Connor, Ana Barabash, Maria Paz de Miguel, Angel Diaz-Perez, Clara Marcuello, Cristina Familiar, Inmaculada Moraga, Maria Arnoriaga-Rodriguez, Johanna Valerio, Laura del Valle, Veronica Melero, Mirella Zulueta, Leire Mendizabal, María Jose Torrejon, Miguel Angel Rubio, Pilar Matia-Martín, and Alfonso Calle-Pascual

Figure S1. Strength Manhattan Plot of Low-Birth-Weigth GWA Functional Enrichment provided by STRING 12.

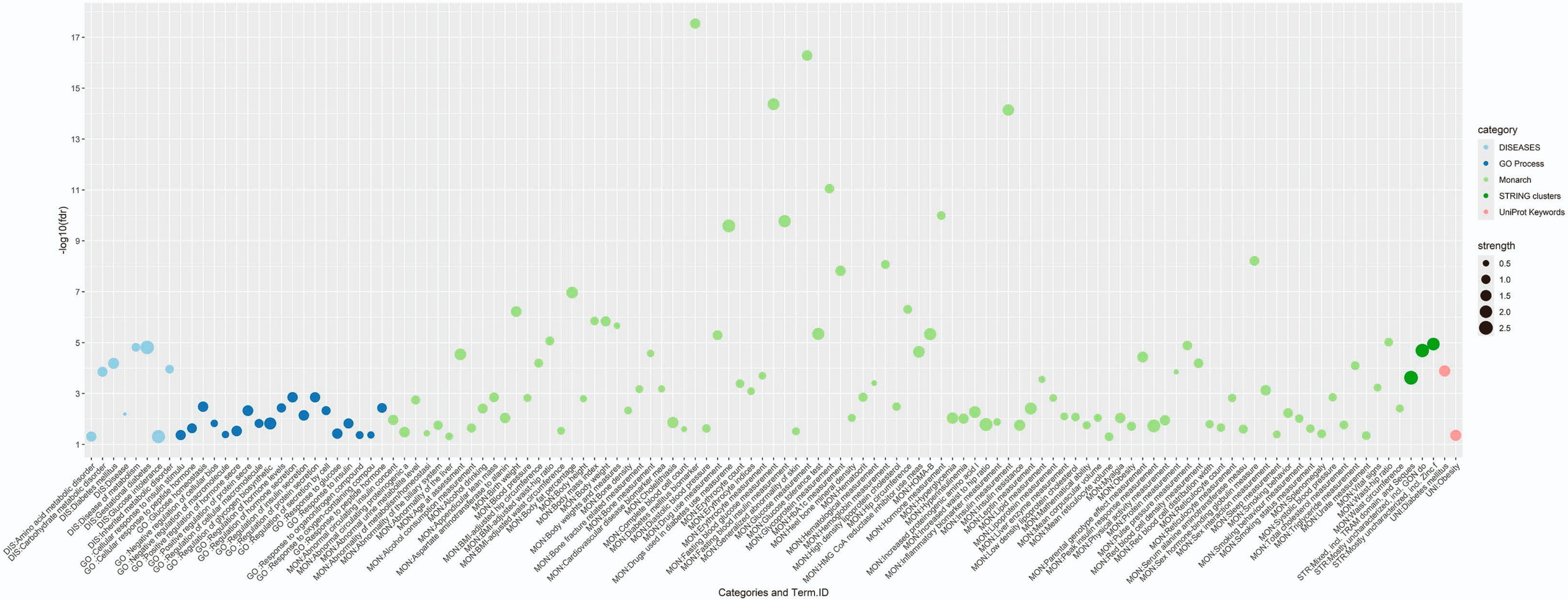

The x-axis includes all functional enrichment terms provided by STRING 12. Size of the point indicates the strength of the association reflected in Table S6.C
